# Oxidative status and metabolic profile in a long-lived bird preparing for extreme endurance migration

**DOI:** 10.1038/s41598-019-54057-6

**Published:** 2019-11-26

**Authors:** Jorge S. Gutiérrez, Pablo Sabat, Luis E. Castañeda, Carolina Contreras, Lucas Navarrete, Isaac Peña-Villalobos, Juan G. Navedo

**Affiliations:** 1 Estación Experimental Quempillén, Facultad de Ciencias, Universidad Austral de Chile, Ancud, Chiloé Chile; 2 Conservation Biology Research Group, Department of Anatomy, Cell Biology and Zoology, Faculty of Sciences, University of Extremadura, Badajoz, Spain; 3 Departamento de Ciencias Ecológicas, Facultad de Ciencias, Universidad de Chile, Santiago, Chile; 4 Center of Applied Ecology and Sustainability, Pontificia Universidad Católica de Chile, Santiago, Chile; 5 Programa de Genética Humana, Facultad de Medicina, Instituto de Ciencias Biomédicas, Universidad de Chile, Santiago, Chile; 6 Bird Ecology Lab, Instituto de Ciencias Marinas y Limnológicas, Universidad Austral de Chile, Valdivia, Chile

**Keywords:** Animal migration, Ecology, Ecophysiology

## Abstract

The high metabolic activity associated with endurance flights and intense fuelling of migrant birds may produce large quantities of reactive oxygen species, which cause oxidative damage. Yet it remains unknown how long-lived birds prepare for oxidative challenges prior to extreme flights. We combined blood measurements of oxidative status and enzyme and fat metabolism in Hudsonian godwits (*Limosa haemastica*, a long-lived shorebird) before they embarked on non-stop flights longer than 10,000 km during their northbound migrations. We found that godwits increased total antioxidant capacity (TAC) and reduced oxidative damage (TBARS) as the pre-migratory season progressed, despite higher basal metabolic rates before departure. Elevations in plasma *β*-hydroxybutyrate and uric acid suggest that lipid and protein breakdown supports energetic requirements prior to migration. Significant associations between blood mitochondrial cytochrome-c oxidase and plasma TAC (negative) and TBARS (positive) during winter indicate that greater enzyme activity can result in greater oxidative damage and antioxidant responses. However enzyme activity remained unchanged between winter and premigratory stages, so birds may be unable to adjust metabolic enzyme activity in anticipation of future demands. These results indicate that godwits enhance their oxidative status during migratory preparation, which might represent an adaptation to diminish the physiological costs of long-distance migration.

## Introduction

Among the most extreme examples of physical performance in the animal kingdom are those of migratory birds, which make some of the most spectacular long-distance migrations of any organism on the planet^1,2^. In birds, the greatest endurance migrations are found among long-distance migratory shorebirds (Charadriiformes), with several members making annual journeys exceeding 30,000 km, including one or more non-stop trans-oceanic flights of 8000–12,000 km^3^. These powered flapping flights of up to 9 days duration, fuelled entirely from stored nutrients and sustained at very high metabolic rates, extend the limits of what is known of endurance physiology^1^.

Intense and prolonged physical activity is normally associated with an increased production of reactive oxygen species (ROS), which cause oxidative damage to lipids, DNA and proteins^4,5^. While there is no one-to-one correspondence between ROS production and oxygen consumption, ROS production rates are expected to be substantially higher in animals with inherently high rates of oxygen consumption, particularly birds^6,7^. Therefore, oxidative stress (i.e. the accumulation of oxidative damage) might occur in migrating individuals as a result of increased ROS production linked to the high metabolic rates that accompany migration^6,8^. Unlike other vertebrates, birds rely primarily on fats (some of which are highly susceptible to attack by ROS) to fuel endurance migration, and thus face a greater potential for damage from the reactive by-products of their own metabolism^8^. Accordingly, some studies have shown that migrating birds can experience oxidative damage during long-duration flights^9–12^. For example, Jenni-Eiermann *et al*.^11^ found that European robins (*Erithacus rubecula*) experience oxidative damage during migratory flights and increase antioxidant capacity. Likewise, Skrip *et al*.^12^ found that fat stores positively correlated with circulating antioxidant capacity in blackpoll warblers (*Setophaga striata*) and red-eyed vireos (*Vireo olivaceus*) preparing for fall migration on Block Island, USA, but were uncorrelated in Italian garden warblers (*Sylvia borin*) after crossing the Sahara Desert and Mediterranean Sea. Together, these studies provide evidence that birds on stopover can prepare for, and recover from, oxidative challenges posed by migratory flights. However, most of the work has been done on passerine species which do not embark on extreme migration (i.e. non-stop flights >5000 km^1^). Furthermore, passerines are short-lived birds that usually compromise oxidative defences when faced with costly activities such as increased breeding effort^13^. The ‘oxidative stress theory of ageing’^5^ predicts that long-lived animals will have less cumulative oxidative damage together with structural characteristics that make them more resistant to oxidative damage itself. Indeed, a recent comparative study showed that bird species with longer lifespan have higher non-enzymatic antioxidant capacity and suffer less oxidative damage to their lipids^14^. Long-lived birds, such as shorebirds^3^, should thus exhibit higher oxidative defences in response to increased migration workload and thus ROS production.

Besides the oxidative cost caused by prolonged flights, migratory birds are also expected to experience oxidative damage during migratory fuelling^15–17^, as this typically involves increases in metabolism and caloric intake^18^. Long-distance migratory shorebirds accumulate large amounts of fat before take-off on migratory flights^19,20^. Given the importance of fats to migrant birds, circulating lipid metabolites are often used to assess individual energetic state. Overall, some lipid metabolites increase during fat deposition (triglycerides), whereas others increase during fat catabolism (glycerol and *β*-hydroxybutyrate)^21–23^; plasma uric acid can also increase due to the deamination of dietary protein for storage as fat or the catabolism of body protein during fasting^21–23^. Despite this, the metabolic correlates of long-distance travel in free-living migrants are as yet poorly studied in the context of oxidative stress^12^.

Here we integrated blood-based measurements of energy metabolism (lipid metabolites, mitochondrial enzyme activity) with oxidative status (oxidative damage and antioxidant capacity) and metabolic rate to examine how Hudsonian godwits (*Limosa haemastica*; hereafter “godwits”) prepare for oxidative challenges posed by extreme endurance migration. Individual godwits make non-stop flights of longer than 10,000 km during their northbound migrations from southern South America to Arctic and sub-Arctic Alaska and Canada^24^. Moreover, godwits can live up to 30 years or longer^25^ and thus provide an excellent model in which to study the link between oxidative stress and self-maintenance in a migratory context. We compared measurements of godwits sampled at three stages before embarking on their northbound migration. We hypothesized that godwits readying for departure would undergo preparation for oxidative challenges posed by extreme endurance migration. Specifically, we predicted (*i*) that fuelling and pre-departure individuals would increase their antioxidant capacity in anticipation of flight demands; (*ii*) that they would show strong correlations between lipid metabolites/fat scores and circulating non-enzymatic antioxidants; and (*iii*) that enzyme activity would correlate with metabolic rate and oxidative damage.

## Methods

### Study system and data collection

**Figure 1:**
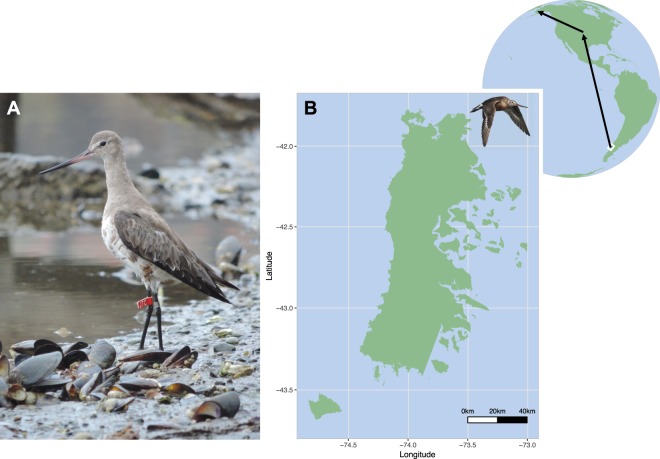
(**A**) Hudsonian godwit overwintering on Chiloé Island (copyright permitted by Juan G. Navedo). (**B**) Map showing Chiloé Island and the northbound migration route of the godwit’s local population (adapted from Senner *et al*. 2014). Map was created with R version 3.5.1.

The study was carried out on a population of Hudsonian godwits (Fig. 1A) that spend the boreal winter on Chiloé Island, southern Chile^26^. Godwits depart from Chiloé Island in March and fly non-stop to central United States, with most individuals stopping only once before again flying non-stop to their breeding grounds in Alaska^24^. Individual godwits make non-stop flights of longer than 10,000 km and 7 days during their northbound migrations^24^ (Fig. 1B). Godwits were caught close to the high-tide roosts (incoming tides) using cannon nets from January to March 2018, spanning three pre-migration stages (based on body mass, fat scores and departure dates on Chiloé from 2014–2018; J.G. Navedo, unpublished data): ‘wintering’ (early January), ‘fuelling’ (mid-February), and ‘pre-departure’ (mid-March). Time elapsed from capture to high tide peak averaged 41 min (range 20–76 min). The potential effect of tide-enforced fasting on metabolite levels^22^ was probably small since godwits typically extend foraging activity up to 2.5 h after the low tide peak^26^ and their retention time (i.e. the average time for food to pass a bird’s digestive tract) is about 1.5 h^27^. Metabolite levels may also change during the time elapsed between capture and blood sampling. Birds were therefore blood-sampled as soon as possible after capture (mean 44 min, range 35–52 min). Approximately 1 ml blood was taken from the right jugular vein and stored within heparinized Eppendorf tubes maintained at 4 °C for up to 3 h. Next, tubes were centrifuged at 10,000 rpm (relative centrifugal force = 9,250) for 10 min, plasma was separated from red blood cells and frozen at −80 °C until analysis. Samples were collected from 54 adults at wintering (*n* = 14:6 females and 8 males), fuelling (*n* = 19:11 females and 8 males), and pre-departure (*n* = 21:11 females and 10 males) stages. Time from capture to bleeding (hereafter “bleed time”) was used in analyses to determine whether there was an effect of time until bleeding on oxidative status and metabolite profiles. Upon capture, birds were also ringed, measured (body mass and standard morphometrics), aged (adult or juvenile), sexed (first based on relative bill length and later through molecular sexing), and had their fat depots scored^28^.

### Scaled mass index

Godwits are sexually dimorphic in size and their mass is highly variable^25^. Thus, we calculated scaled mass index (SMI) for males and females separately following Peig & Green^29^. This method normalizes body mass to a fixed value of body size based on the scaling relationship between mass and length measures^29^. The fixed value of body size was the mean value of wing length for the study population: 220.8 ± 0.5 mm (*n* = 129) for females and 210.8 ± 0.4 mm (*n = *208) for males (J.G. Navedo unpublished data).

### Oxidative status

Lipid peroxidation in red blood cells was measured by the thiobarbituric acid test (thiobarbituric acid-reactive substances, TBARS), which relies on the ability of polyunsaturated fatty acids of cell membranes to readily react with ROS by donating a hydrogen atom^30^. Briefly, lipid peroxidation was measured using a commercial kit (Oxiselect, STA-330 Cell biolabs). The assay evaluates the joint between Malondialdehyde (MDA, a product of lipid peroxidation) and thiobarbituric acid. Absorbance was monitored in a Thermo Scientific Multiskan GO UV/VIS spectrophotometer at 25 °C. We determined hydrogen peroxides in plasma (H_2_O_2_, a major form of ROS) as a measure of the pro-oxidant status^4^ using a commercial kit (MAK311, Sigma Aldrich). The assay evaluated colorimetrically the oxidation of Fe^+2^ to Fe^+3^ by peroxides present in the sample at 585 nm.

We estimated non-enzymatic total antioxidant capacity (TAC) by measuring the capacity of a plasma sample to quench a standardized free radical challenge, following Sabat *et al*.^31^. Briefly, TAC was measured by colorimetric reaction using a commercial assay kit (Sigma Aldrich, San Diego, CA, #CS0790). The assay is based on the formation of a ferryl myoglobin radical from metmyoglobin and H_2_O_2_ which oxidizes the ABTS (2,2′-azino-bis(3-ethylbenzthiazoline-6-sulfonic acid)) to form the radical ABTS•+ which in turn produces a chromogen that can be detected spectrophotometrically at 405 nm (Multiskan GO). This protocol used the Trolox, a Vitamin E analog to be compared with the antioxidant capacity in a sample. As pointed out by Cohen *et al*.^32^ this method is a functional measure of antioxidant capacity that includes the action of micromolecular components such as vitamins E and C, and uric acid among others. Because uric acid has antioxidant properties and can reflect an adaptive response to oxidative stress^33^, we determined plasma uric acid concentrations (see below). Finally, we calculated oxidative balance as the ratio of TBARS and TAC (×1000) to generate a single variable (TBARS:TAC) that captured interindividual variation in oxidative balance (with higher values meaning higher oxidative stress^9^).

### Mitochondrial enzyme activity

The enzyme activities of cytochrome c oxidase (COX, E.C. 1.9.3.1) and citrate synthase (CS, E.C. 4.1.3.7) were measured in mitochondrion of red blood cells as a proxy of metabolic intensity of other tissues, such as skeletal muscle^34^. COX is involved in the last reaction of the mitochondrial respiratory chain that is indicative of the energy capacity of the mitochondrial system, whereas CS catalyses the reduction of oxygen to water^35^. Besides, these metabolic enzymes in skeletal muscle are functionally associated with oxygen consumption (e.g., metabolic rate, see below) at the organismal level in birds^31,35,36^. COX and CS activities were measured using the methods described in Sabat *et al*.^31,37^. Enzyme activities are reported as specific activity per gram of protein (µmol min^−1^ mg protein^−1^). Protein concentration in plasma and red blood cell homogenate was determined after Bradford^38^ using bovine serum albumin as standard.

### Plasma metabolites

We performed all metabolite assays at the Laboratorio Clínico Veterinario (Universidad Austral de Chile) using an auto-analyzer (Metrolab 2300, Wiener Lab). Briefly, total triglycerides (triglycerides plus free glycerol) was measured by enzymatic colorimetric assay (Triglycerides liquicolormono GPO-PAP kit, HUMAN GmbH) adapted to small sample volumes in 300 μL flat-bottom microplates. Glycerol concentration was determined by a coupled enzyme assay (Sigma-Aldrich MAK117). Concentration of uric acid was determined using the enzymatic colorimetric test with lipid clearing factor (uric acid liquicolor PAP kit, HUMAN GmbH). *β*-hydroxybutyrate was measured using colorimetric enzymatic reaction (D-3-hydroxybutyrate kit; Ranbut, Randox Laboratories). Triglyceride levels were calculated by subtracting free glycerol from total triglyceride levels^23,39^.

### Metabolic rate measurements

A subsample of 18 adult birds was taken into nearby bird facilities at Quempillén Experimental Station for metabolic rate measurements. Due to logistical limitations we were not able to measure metabolic rates during the wintering period, so we measured the metabolic rates of fuelling (*n* = 10) and pre-departure (*n* = 8) godwits. Birds were housed in indoor aviaries (5 × 2.5 × 2.5 m) with access to freshwater and fasted for at least 6 h prior to metabolic trials the same night^40^. BMR was measured as the overnight oxygen consumption ($\dot{V}$O_2_, ml min^−1^) with a Field Metabolic System (FMS, Sable Systems, Las Vegas, NV). Birds (four individuals per night) were placed into adjacent metabolic chambers (15 L) in complete darkness and located in a temperature-controlled room at a constant temperature of 26 °C, i.e. within the thermoneutral zone^40^. Incurrent ambient air at a controlled flow rate of 10,000 mL min^−1^ was pumped through needle valves (air flow manifold MF-8; Sable Systems, Las Vegas, NV), which supplied five chambers with a constant flow (1400–2000 mL min^−1^). Excurrent air was subsampled at 200 mL min^−1^ and passed through a hygrometer, a CO_2_ analyser, and a fuel cell O_2_ analyser (i.e. the FMS). The O_2_ analyser was calibrated before each trial using 99.995% pure N_2_ as the low reference and ambient air scrubbed of water vapour and CO_2_ (set to 20.95% O_2_). The CO_2_ analyser was calibrated daily using pure N_2_ as the low reference and a certified mixture of 1.01% CO_2_ as the high reference. The hygrometer was calibrated weekly according to recommendations of the manufacturer. We used a multiplexer to monitor each chamber for 20 min before switching to the next chamber. A 10-min baseline of reference airflow, provided by an empty chamber of the same size, was measured after each round of recordings. All data were recorded using an analog-to-digital converter (UI-2; Sable Systems, Las Vegas, NV) connected to a laptop computer. We corrected for gas analyser drift and lag time of the respirometry system using ExpeData software. Main flow rate was corrected to standard temperature and pressure using eq. 8.6 in Lighton^41^. We did not scrub water vapour before gas analysis during measurements, but corrected for this dilution effect during data analysis using eq. 15.3 in Lighton^41^. $\dot{V}$O_2_ and CO_2_ production rate ($\dot{V}$CO_2_) were calculated using eqs. 11.7 and 11.8 in Lighton^41^, respectively. We considered BMR as the lowest 5-min average $\dot{V}$O_2_ over the test period. Finally, we calculated the respiratory quotient (RQ, i.e. the ratio of $\dot{V}$CO_2_ to $\dot{V}$O_2_). RQ varies from 0.7 (pure lipid catabolism) to 1.0 (pure carbohydrate catabolism); a mix of lipid and carbohydrate catabolism yields intermediate values^41^. BMR measured in the absence of CO_2_ absorbents gives a measure of the non–carbohydrate-fuelled metabolism of the animal, which can provide interesting data to demonstrate a shift in the catabolic allocation of respiratory substrates^41^. The body mass reported for BMR analysis was taken to be the mean of the initial measurement and the final measurement.

### Statistical analyses

To test for temporal effects on physiological variables, we fitted separate general linear models (GLMs) with each trait measure in turn as the response variable. The full model included all three-way interactions between the fixed effects of pre-migration stage (wintering, fuelling, and pre-departure), sex, mass (either mass or SMI), and bleed time. For BMR analysis, GLM included stage (fuelling vs. pre-departure), sex, and mass. Model simplification was conducted using an information theoretical approach^42^. Because the weights of the ‘best’ models (lowest AIC) were always <0.9 (Appendix S1), we used model averaging to identify the most important predictor variables^43^. Relative importance analysis was carried out with the model-averaging approach^42^. In this way, we obtained model-averaged parameter estimates that were directly comparable to each other. We estimated the parameters from the set of all models for which the sum of Akaike weights reached >0.95. We considered predictor variables that had model-averaged 95% confidence intervals (CIs) that did not cross zero to be biologically relevant (i.e. significant). Results were qualitatively similar when using either body mass or SMI as predictor variable (Appendix S1). For simplicity, here we only present results from analyses using body mass. We ran all models using the R (version 3.5.1, R Core Team 2016) base function ‘glm’ and the *MuMIn* package for model averaging^44^.

To test for relationships between oxidative and metabolic variables, we ran regression analyses for the whole study period and for each stage separately. Predictor and response variables were log transformed when necessary to meet normality assumptions.

#### Ethics statement

All experimental procedures were carried out under the approval of the Bioethics Subcommittee of Universidad Austral de Chile (no 260/2016). All methods were carried out in accordance with these approved guidelines and regulations.

## Results

### Oxidative status

**Figure 2:**
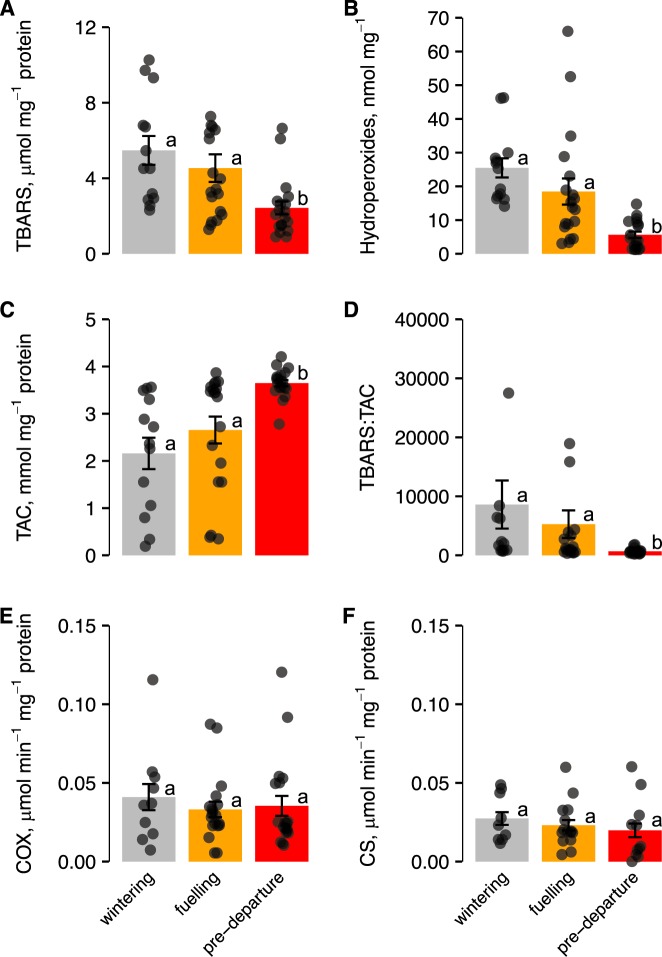
Levels of oxidative stress markers (**A–D**) and enzyme activities (**E,F**) in Hudsonian godwits at different pre-migration stages. Data are means ± s.e.m. Different letters indicate differences between stages based on 95% CIs. Note different scales on the y axes.

Levels of TBARS were lower at pre-departure (*β* = −2.88, s.e.m. = 1.110, CI = −5.11, −0.65), compared to wintering and fuelling stages (Fig. 2). Stage was the only important predictor for TBARS, having 71% relative importance. Likewise, H_2_O_2_ levels were lowest at pre-departure stage (*β* = −14.79, s.e.m. = 6.81, CI = −28.13, −1.46) (Fig. 2); stage had 73% relative importance. Conversely, TAC and uric acid concentrations were substantially higher at pre-departure stage (TAC: *β* = 1.48, s.e.m. = 0.44, CI = 0.60, 2.35; uric acid: *β* = 443.34, s.e.m. = 182.22, CI = 78.92, 807.76; Fig. 2), and stage was again the only important predictor variable for both TAC and uric acid, having 99% and 98% relative importance, respectively. Finally, TBARS:TAC was lowest at pre-departure (*β* = −1.62, s.e.m. = 0.49, CI = −2.61, −0.63; Fig. 2), indicating that levels oxidative stress were lowest at this stage. In this case stage was again the only clear significant predictor variable, having 96% relative importance.

### Mitochondrial enzyme activity

**Figure 3:**
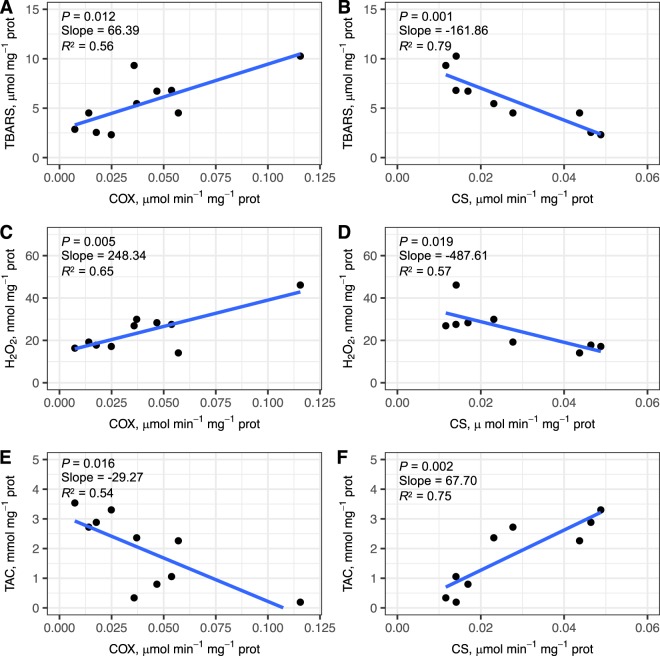
Relationships between (**A**) TBARS and COX; (**B**) TBARS and CS; (**C**) H_2_O_2_ and COX; (**D**) H_2_O_2_ and CS; (**E**) TAC and COX; and (**F**) TAC and CS in Hudsonian godwits during the wintering period. Lines represent linear regressions.

There was no support for temporal (stage) and intrinsic (sex and body mass) effects on enzyme activity measures (all predictor variables had 95% CIs that crossed zero; Fig. 2 and Appendix S1). At the wintering stage, however, there were significant and positive associations between COX activity and oxidative damage (TBARS and H_2_O_2_), as well as a significantly negative association between COX activity and TAC (Fig. 3). In contrast, CS correlated negatively with TBARS and H_2_O_2_, and positively with TAC during the winter stage (Fig. 3). Neither COX nor CS activities were significantly correlated to oxidative status at the fuelling and pre-departure stages (always *P* > 0.05, Appendix S2).

### Lipid metabolites and fat scores

**Figure 4:**
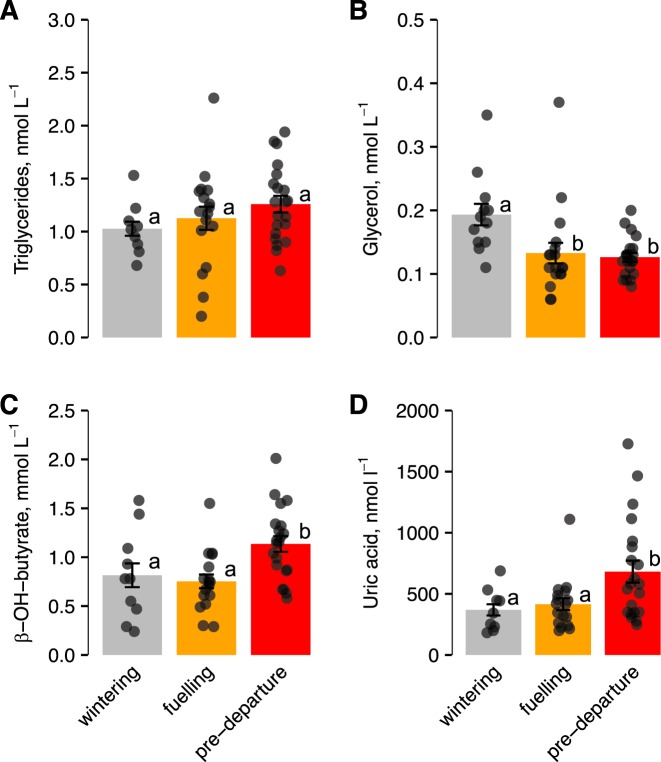
Levels of plasma metabolites in Hudsonian godwits at different pre-migration stages. Data are means ± s.e.m. Different letters indicate differences between stages based on 95% CIs. Note different scales on y axes.

Plasma *β*-hydroxybutyrate concentrations peaked during pre-departure (*β* = 0.48, s.e.m. = 0.19, CI = 0.10, 0.85; Fig. 4); stage was the only significant predictor variable for *β*-hydroxybutyrate, with a relative importance of 100%. In contrast, glycerol decreased with time, with concentration being lower at the fuelling (*β* = −0.057, s.e.m. = 0.02, CI = −0.10, −0.017) and pre-departure stages (*β* = −0.06, s.e.m. = 0.02, CI = −0.11, −0.02) (Fig. 4). None of the predictor variables had a strong effect on triglyceride concentrations (95% CIs crossed zero; Fig. 4). However, triglyceride concentrations correlated positively with fat scores (*F*_1,45_ = 4.42, *P* = 0.041). Fat scores were highest at pre-departure, particularly in males (GLM: stage(pre-departure): *β* = 3.83, s.e.m. = 0.58, CI = 2.69, 4.97; stage(pre-departure) × sex(male): *β* = 1.99, s.e.m. = 0.80, CI = 0.43, 3.55).

### Metabolic rate measurements

**Figure 5:**
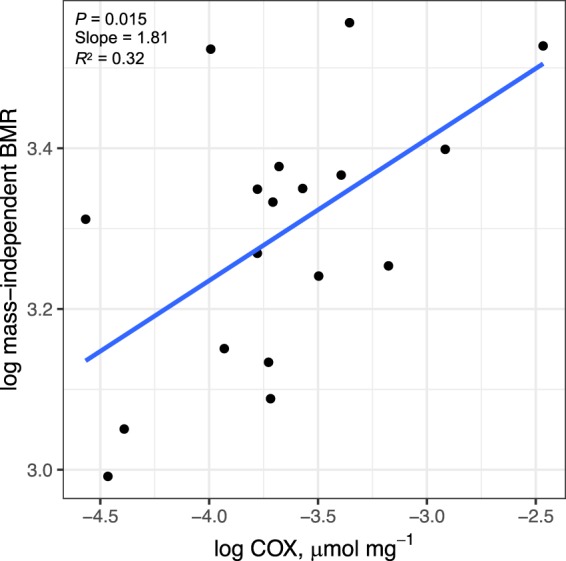
Relationships between mass-independent BMR and COX activity (fuelling and pre-departure data pooled). Line represents linear regression.

BMR was higher in pre-departure godwits (*β* = 1.54, s.e.m. = 0.70, CI = 0.07, 3.00) than in fuelling godwits; stage had a relative importance of 65%. Overall body mass had a positive, significant effect on BMR (*β* = 0.03, s.e.m. = 0.01, CI = 0.002, 0.050, with a relative importance of 82%). Yet the interaction ‘body mass × sex’ (*β* = −0.05, s.e.m. = 0.02, CI = −0.09, −0.02) indicated that body mass had contrasting effects on BMR in the two sexes: BMR was positively correlated with body mass in females but the trend was negative in males. When using linear regression analysis between BMR and body mass, irrespective of sex and stage, values of BMR and body mass were significantly and positively correlated (*F*_1,17_ = 6.59, *P* = 0.021). Therefore, we used mass-independent BMR (residuals from its regression against body mass) to test for potential relationships between BMR and enzyme activities. Mass-independent BMR correlated positively with COX activity (*F*_1,16_ = 7.46, *P* = 0.015; Fig. 5), but not with CS activity (*F*_1,11_ = 0.81, *P* = 0.39). Neither whole-animal nor mass-independent BMR correlated with any markers of oxidative status (always *P* > 0.05). RQ did not differ between fuelling (0.74 ± 0.03) and pre-departure (0.76 ± 0.04) godwits (*t*_1,17_ = 1.34, *P* = 0.201), and did not correlate significantly with fat stores, lipid metabolites, and oxidative damage (always *P* > 0.05).

## Discussion

We showed that Hudsonian godwits prepare for extreme non-stop flights on their ‘wintering’ grounds by simultaneously increase antioxidant capacity and reduce oxidative damage, despite the intense fuelling and high metabolic rates accompanying the pre-migration period. If considering the context of birds on stopover^8^, it is no surprise that godwits increase antioxidants as they build fat stores. Yet one might also expect them to experience oxidative damage during fuelling^15,16^. Instead, godwits decreased oxidative damage before take-off, which resulted in a more positive effect on their oxidative status. This likely represents an adaptation to diminish the oxidative costs during periods of flight and refuelling^17^. Moreover, these results support the notion that long-lived species are more resistant to oxidative damage than short‐lived ones^14^. Godwits are likely to prioritize self-maintenance, as shown by their increased antioxidant capacity, and this may underlie the pronounced longevity recorded in this and other long-distance migratory shorebirds^3,25^.

The positive trend in antioxidant capacity with time likely reflects the abundance of antioxidant-rich food resources on Chiloé. A recent study indicates that shorebird populations encounter a predictable and abundant food supply on the intertidal mudflats of Chiloé^45^. Hudsonian godwits mainly feed on polychaetes^46,47^, which could act as a source of antioxidants^48^. Importantly, dietary antioxidants can supplement or replace the action of endogenous components of the antioxidant system^49^. Thus, an increase in the intake of antioxidant-rich food during the pre-migratory period^26^ — irrespective of whether birds preferentially select food rich in antioxidants or antioxidant resources come from a variety of food items available in their natural habitat — is likely to have reduced oxidative damage in pre-departure godwits. As pointed out by Beaulieu & Schaefer^50^, migratory birds may use specific antioxidants in anticipation of impending need. This strategy could be important for godwits as they may be unable to ameliorate any damage during non-stop flights. Furthermore, the risk of excessive flight muscle catabolism increases with the duration of non-stop flights^11^, a situation regularly faced by godwits *en route*^19,20^. Thus, godwits must heavily invest in antioxidant defences against the consequences of flight muscle catabolism and resulting oxidative damage.

Additionally, plasma concentrations of three key metabolites varied among stages. The elevation in plasma *β*-hydroxybutyrate (a ketone body synthesized from free fatty acids) prior to endurance flight may replace part of the glucose during short-term fasting^22,51^. Previous work with Bar-tailed godwits (*Limosa lapponica*) subjected to inactive fasting during stopover showed a trend to increase plasma butyrate^22^, suggesting that it supports general energetic requirements rather than the specific energetic demands of flight. It is therefore possible that godwits subjected to short-term, tide-enforced fasting increased plasma *β*-hydroxybutyrate temporarily. However, the time elapsed from capture and high tide peak (roosting) was similar across stages, and thus unlikely to have caused seasonal differences in levels of *β*-hydroxybutyrate and other metabolites. An alternative explanation is that godwits readying for departure had started mobilizing lipids. This is likely as pre-departure godwits had reached peak fat scores and the numbers of godwits on Chiloé dropped in mid-March (when they were captured). Elevated levels of *β*-hydroxybutyrate reduce glucose utilization and play an important role in the sparing of carbohydrate and protein^52^. Consequently, the plasma *β*-hydroxybutyrate levels of pre-departure godwits were increased compared with wintering and fuelling godwits. This is also consistent with the finding that elevated *β*-hydroxybutyrate in semipalmated sandpipers (*Calidris pusilla*) at stopover areas reflects slower weight gain prior to non-stop flight to the breeding grounds^23^. In contrast, glycerol concentrations were lower in birds preparing for migration, compared to those of overwintering ones. Glycerol is produced by lipolysis of triglycerides in adipose tissue and muscle during periods of negative energy balance and exercise^53^. Fuelling and pre-departure godwits were likely in positive energy balance, albeit the higher metabolic rates before flight. Thus, glycerol levels may have decreased due to high rates of fat deposition. This is consistent with a study with captive western sandpipers (*Calidris mauri*) showing that plasma glycerol was negatively related to mass gain^54^.

The significant increase in uric acid levels at the pre-departure stage likely indicates that birds increased their intake of dietary protein prior to endurance flight, given that uric acid is the final product of protein catabolism in birds and correlates closely with protein consumption^55^. Nonetheless, higher uric acid concentrations in pre-departure godwits could have also resulted from the breakdown of proteins that originate from body tissue. This seems to be the case for other birds that change body composition while preparing for a migratory flight^20,56,57^, suggesting a common pattern in allocation of lean body mass prior to endurance flight. Notably, bar-tailed godwits preparing to depart exhibit a reduction in size of digestive tissues, possibly to fly with a total mass as low as possible^19,20^. Overall, changes in plasma *β*-hydroxybutyrate and uric acid concentrations suggest that during the premigratory period energy expenditure entails catabolism of lipids and proteins rather than a shift in fuel substrate. Indeed, the similar RQ in fuelling and pre-departure individuals indicates that they used predominantly, but not solely, fat as fuel. Unfortunately we did not measured BMR in overwintering birds, so we do not know whether they increased their BMR throughout the entire pre-migratory period. However, pre-departure godwits had higher BMR than fuelling godwits, probably reflecting changes in the size or metabolic activity of different organs and tissues associated with migratory disposition^18,58^. Interestingly BMR increased with mass in females, but not in males. Sexual dimorphism in BMR has been reported for several bird species^59,60^. Such sex differences in the relationships between BMR and body mass may result from between-sex differences in metabolically inactive tissues such as fat (males had larger fat scores than females at pre-departure) and/or differences in the amount of energy required for energetically demanding tasks, such as migration and reproduction^18,59^.

Although the relationship between the rate of oxygen consumption and the generation of ROS is currently unresolved^61–63^, some studies have found support for a functional relationship between mass-specific metabolic rates and oxidative status^31,64^. For example, Sabat *et al*.^31^ showed that increased salt intake elicited changes in the BMR of Rufous-collared sparrows (*Zonotrichia capensis*), which were in turn coupled with an increase in the activity of mitochondrial enzymes and changes in oxidative status. Although we did not find a significant relationship between mass-independent BMR and oxidative status, we found that mass-independent BMR correlated positively with COX activity in mitochondria from red blood cells, suggesting that the rate of oxygen consumption is to some extent driven by tissue-specific metabolic demands. This supports the notion that avian erythrocytes possess functional mitochondria in terms of respiratory activities and ROS production^65,66^. Moreover, mitochondrial parameters (such as mitochondrial O_2_ consumption, the capacity of the electron transport system and the capacity to produce ATP via oxidative phosphorylation) measured in red blood cells correlated to those measured in the pectoral muscle in free-ranging king penguins (*Aptenodytes patagonica*)^34^. Hence, measures of mitochondrial function in red blood cells may reflect what is happening in other tissues (e.g., pectoral muscle), and thus provide general information at the organismal level^34^. This warrants further investigation since blood enzyme activities could represent informative non-invasive markers for monitoring the rate of energy expenditure in free-ranging birds, only requiring a small volume (~30 μl) of blood. In addition, we found that TBARS increased, yet TAC decreased, with COX activity during the wintering period. This finding indicates that greater enzyme activity can result in greater oxidative damage outside the migration period. Conversely, we found the opposite pattern for CS. The reason of this contradictory result is puzzling. A recent study^67^ found CS activity in blood cells of chicks of yellow-legged gull (*Larus michahellis*) was negatively correlated with mtDNA damage, indicating a negative relationship between oxidative damage and mitochondrial activity. Nevertheless, biochemical variables and oxidative damage in gull chicks were also correlated with growth rate. Thus, we cannot rule out that the negative association between CS and oxidative damage found in overwintering godwits was caused by other physiological processes (such as tissue growth and repair) occurring in parallel. It is important to note that blood enzyme activities did not increased prior to endurance flight. Along this line, adult western sandpipers ready to migrate did not alter flight muscle catabolic enzymes (including CS activity) from earlier in the winter, suggesting that modulation of several physiological characteristics occur after migratory movements began^68^. Likewise, the levels of CS in semipalmated sandpipers preparing to migrate non-stop from eastern Canada to wintering sites on the northwest coast of South America in late summer do not change before their transoceanic flight, suggesting that changes in body composition can occur without an increase in mitochondrial enzymes^69^. Together, these findings imply that before spring migration, different shorebirds may be unable to adjust their metabolic enzyme activity in anticipation of future demands^68^.

Our findings provide an exciting opportunity for future work to test the hypothesis that high levels of ROS will drive potential long-term costs of oxidative stress in animals responding the challenges of a demanding migration, which must be scheduled optimally around other activities such as reproduction, moult, and territory acquisition^3^. To better understand the oxidative costs and defences, one important challenge in the future will be to determine how oxidative status may affect timing of migration and thus reproduction. Further studies should also examine how this physiological parameter is balanced between parents and offspring^70^. In this context, a longitudinal approach examining the oxidative status of organisms throughout the different stages of their annual cycle will be valuable. In the face of rapid and extensive human-induced environmental change, understanding how migratory animals accrue adequate energy stores and regulate the levels of pro-oxidants and antioxidants have far-reaching implications for evolutionary and population ecology, as well as for animal conservation and related policy decisions.

In conclusion, our study shows that trans-hemispheric migrant birds can increase circulating antioxidant capacity and reduce oxidative damage in anticipation of extreme non-stop flights. Our results indicate that godwits do not experience greater levels of oxidative stress during intense fuelling. They also suggest that alleviation of the accumulation of oxidative damage seems to start early in the pre-migration season, which may be a selective advantage for individuals that have higher rates of aerobic respiration. While extreme non-stop migration can represent an oxidative challenge for animals, protective mechanisms like increasing antioxidant defences and decreasing oxidative damage seem to occur in Hudsonian godwits. This might represent an adaptation to diminish the physiological costs of endurance migration.

## Supplementary information

Supplementary information
