# Supplementary material for: Oxidative status and metabolic profile in a long-lived bird preparing for extreme endurance migration
Source: Sci Rep. 2019 Nov 26;9:17616. doi: 10.1038/s41598-019-54057-6 (PMC6879648; doi:10.1038/s41598-019-54057-6)

Supporting Information to 'Oxidative status and metabolic profile in a long-lived bird preparing for extreme endurance migration'

Jorge S. Gutiérrez, Pablo Sabat, Luis E. Castañeda, Carolina Contreras, Lucas Navarrete, Isaac Peña-Villalobos, Juan G. Navedo

Appendix S1

Table S1. Top candidate models explaining variation in oxidative status; mitochondrial enzyme activity, BMR, and plasma metabolites in Hudsonian godwits captured at three pre-migration stages (winter, fuelling, predeparture). AIC_c, Akaike information criterion for small sizes; ΔAIC_c, difference compared with the top model; ω, model weighting.

Functional group: measure	log-likelihood	AIC_c	ΔAIC_c	ω	residual
**Oxidative status: TBARS (damage)**					
stage	-119.586	248.0	0.00	0.249	324.93
stage + sex	-119.286	249.9	1.86	0.098	321.13
stage + bleed time	-119.295	249.9	1.88	0.097	321.24
**Oxidative status: H_2O_2 (pro-oxidant status)**					
stage + bleed time	-192.080	395.5	0.00	0.122	6357.1
stage + bleed time + mass + mass × bleed time	-189.615	395.9	0.37	0.101	5760.3
bleed time + mass + mass × bleed time	-192.390	396.1	0.62	0.089	8494.8
stage	-193.702	396.3	0.77	0.083	6783.2
**Oxidative status: TAC (antioxidant capacity)**					
stage	-70.759	150.4	0.00	0.322	47.885
stage + bleed time	-70.171	151.7	1.29	0.169	46.794
**Oxidative status: uric acid (antioxidant capacity)**					
stage + mass	-334.368	680.2	0.00	0.188	10.583
stage	-335.628	680.2	0.01	0.187	11.363
stage + mass + bleed time	-333.565	681.2	1.03	0.112	10.387
**Oxidative status: TBARS:TAC**					
stage	-78.761	166.4	0.00	0.350	65.538
stage + bleed time	-78.440	168.2	1.82	0.141	64.717
**Metabolism: COX activity**					
bleed time	-42.204	91.0	0.00	0.399	0.026
bleed time + mass	-42.003	92.9	1.98	0.148	0.025
**Metabolism: CS activity**					
null	95.196	-186.0	0.00	0.200	0.009
bleed time	95.933	-185.1	0.93	0.126	0.009
**Metabolism: BMR***					
mass + sex + mass × sex	-23.131	61.3	0.00	0.331	13.771
mass + stage + sex + mass × sex	-21.100	61.8	0.58	0.248	10.990
mass	-27.602	62.9	1.66	0.145	22.631
**Metabolism: true triglycerides**					
sex	-16.404	39.4	0.00	0.202	5.531
sex + bleed time + sex × bleed time	-14.085	39.6	0.27	0.177	5.011
sex + bleed time	-15.568	40.1	0.72	0.141	5.338
sex + mass	-15.856	40.7	1.30	0.105	5.403
**Metabolism: glycerol**					
stage + sex	81.012	-150.7	0.00	0.368	0.122
**Metabolism: *β*-OH-butyrate**					
stage + bleed time + mass + bleed × mass	-10.648	38.1	0.00	0.279	4.380
stage + bleed time + mass	-12.184	38.4	0.32	0.238	4.670
stage + mass	-14.249	39.9	1.83	0.112	5.089

*Full model included stage (only fuelling and pre-departure), sex, mass (taken before and after BMR measurements), and sex × mass interaction (see *Methods* for details).

Table S2. Summary results for each physiological variable (A-K) after model averaging when using SMI instead of body mass as predictor variable. These results are comparable to those shown in the *Results* section of the main text (i.e. using body mass as predictor variable). Predictor variables in bold had model-averaged 95% confidence intervals (CIs) that did not cross zero. RI = Relative Importance (from 0 to 1). Reference level for stage is 'wintering' (except for BMR, which was only measured at fuelling and pre-departure stages, see below); reference level for sex is 'female'.

(A) TBARS

	Estimate	Unconditional SE	CI (95%)	RI
(intercept)	7.720	4.90	-1.178, 16.619	
StageFuelling	-0.870	0.974	-2.828, 1.087	0.90
**StagePre-departure**	**-2.838**	**0.992**	**-4.832, -0.843**	0.90
SMI	-0.017	0.018	-0.053, 0.019	0.44
SexMale	0.475	1.713	-2.966, 3.916	0.25
Bleed time	-0.023	0.044	-0.111, 0.065	0.36
Bleed time x SMI	0.0004	0.0002	-0.0002, 0.0007	0.07
Bleed time x sex	-0.015	0.018	-0.051, 0.022	0.02
Sex x SMI	0.0005	0.020	-0.042, 0.0427	0.01

(B) H_2O_2

	Estimate	Unconditional SE	CI (95%)	RI
(intercept)	6399	46.92	-28.684, 156.666	
StageFuelling	-3.641	5.083	-13.822, 6.539	1
**StagePre-departure**	**-1.602**	**5.066**	-26.176, -5.861	**1**
SMI	-0.119	0.156	-0.516, 0.098	0.57
SexMale	2.297	7.280	-2.966, 3.916	0.45
Bleed time	-0.335	0.409	-1.256, 0.394	0.78
**Bleed time x SMI**	**9.139e-04**	**1.303e-03**	**1.334e-04, 0.004**	**0.40**
Bleed time x sex	1.400e-03	2.943e-02	-0.195, 0.233	0.07
Sex x SMI	-1.129e-03	1.901e-02	-0.235, 0.166	0.03

(C) TAC

	Estimate	Unconditional SE	CI (95%)	RI
(intercept)	8.266	1.045	6.178, 10.354	
StageFuelling	-0.512	-0.434	-1.384, 0.361	1
**StagePre-departure**	**-1.596**	**0.4387**	**-2.478, -0.714**	**1**
SMI	-7.514e-04	3.189e-03	-0.009, 0.013	0.25
SexMale	5.614e-02	2.526e-01	-1.055, 0.629	0.24
Bleed time	-1.332e-03	6.063e-03	-0.015, 0.009	0.30
Bleed time x SMI	1.381e-06	1.722e-05	-0.0001, 0.0003	0.02
Bleed time x sex	-1.179e-04	1.405e-03	-0.023, 0.010	0.02

(D) Acid uric

	Estimate	Unconditional SE	CI (95%)	RI
(intercept)	54.458	881.638	-1692.076, 1800.993	
StageFuelling	54.009	134.585	-217.314, 325.332	0.88
**StagePre-departure**	**320.162**	**132.894**	**52.409, 587.916**	**0.88**
SMI	3.0714	4.064	-4.999, 11.142	0.39
SexMale	-261.799	1392.370	-3016.179, 2492.582	0.25
Bleed time	4.546	10.549	-16.383, 25.474	0.34
Bleed time x SMI	-0.050	0.040	-0.131, 0.031	0.12
Bleed time x sex	53.008	21.977	8.477, 97.539	0.02

(E) TBARS:TAC

	Estimate	Unconditional SE	CI (95%)	RI
(intercept)	1.933	0.942	0.054 3.812	
StageFuelling	0.448	0.374	-0.304, 1.199	1
**StagePre-departure**	**1.427**	**0.379**	**0.666, 2.188**	**1**
SMI	4.871e-04	2.843e-03	-0.009, 0.013	0.23
SexMale	-5.239e-02	2.277e-01	-1.055, 0.629	0.24
Bleed time	1.843e-03	6.345e-03	-0.014, 0.025	0.36
Bleed time x SMI	-1.873e-06	1.800e-05	-0.0003, 9.694e-05	0.02
Bleed time x sex	1.278e-04	1.343e-03	-0.008, 0.019	0.02

(F) COX

	Estimate	Unconditional SE	CI (95%)	RI
(intercept)	-1.934	1.954	-5.812, 1.945	
StageFuelling	6.013e-04	5.623e-02	-0.474, 0.496	0.05
StagePre-departure	7.450e-03	6.529e-02	-0.359, 0.633	0.05
SMI	-1.909e-03	6.254e-03	-0.009, 0.013	0.49
SexMale	-2.150e-02	3.951e-01	-1.498, 1.357	0.30
Bleed time	2.332e-05	5.250e-05	-4.841e-02, 0.016	1
Bleed time x SMI	1.472e-04	1.238e-03	-3.178e-05, 0.0002	0.24
Bleed time x sex	-4.057e-05	1.316e-03	-1.090e-02, 0.009	0.06

(G) CS

	Estimate	Unconditional SE	CI (95%)	RI
(intercept)	2.571e-02	1.694e-02	-8.519e-03, 5.993e-02	
StageFuelling	-3.121e-04	2.218e-03	-1.823e-02, 1.017e-02	0.08
StagePre-departure	-5.537e-04	2.840e-03	-2.253e-02, 8.225e-03	0.08
SMI	5.876e-06	4.959e-05	-1.814e-04, 2.343e-04	0.22
SexMale	-1.625e-03	5.067e-03	-2.204e-02, 1.086e-02	0.29
Bleed time	-2.308e-05	1.025e-04	-3.799e-04, 2.601e-04	0.39
Bleed time x SMI	1.905e-08	2.883e-07	-5.056e-06, 2.781e-06	0.02
Bleed time x sex	3.039e-06	2.752e-05	-1.432e-04, 3.685e-04	0.03

(H) BMR

	Estimate	Unconditional SE	CI (95%)	RI
(intercept)	3.866	3.988	-4.155, 11.886	
StagePre-departure*	0.218	0.496	-0.625, 2.167	0.28
SMI	0.013	0.013	0.001, 0.043	0.58
SexMale	-0.468	0.711	-2.570, 0.322	0.41

*Reference level = ‘fuelling’

(I) True triglycerides

	Estimate	Unconditional SE	CI (95%)	RI
(intercept)	3.962e-01	7.383e-01	-1.079, 1.871	
StageFuelling	6.211e-03	5.099e-02	-2.449e-01, 3.780e-01	0.09
StagePre-departure	1.459e-02	6.515e-02	-1.515e-01, 4.642e-01	0.09
SMI	1.403e-03	2.293e-03	-2.804e-03, 7.516e-03	0.59
SexMale	3.913e-01	7.560e-01	-1.109, 1.891	1
Bleed time	1.727e-03	4.564e-03	-8.271e-03, 1.364e-02	0.64
Bleed time x SMI	-4.199e-07	1.334e-05	-9.417e-05, 8.471e-05	0.02
Bleed time x sex	-4.027e-04	6.149e-03	2.411e-02, 2.127e-02	0.28

(J) Glycerol

	Estimate	Unconditional SE	CI (95%)	RI
(intercept)	2.213e-01	8.166e-02	5.909e-02, 3.835e-01	
**StageFuelling**	**-5.941e-02**	**2.086e-02**	**-1.006e-01, -1.926e-02**	**0.99**
**StagePre-departure**	**-6.214e-02**	**2.046e-02**	**-1.023e-01, -2.298e-02**	**0.99**
SMI	-1.420e-04	2.458e-04	-8.718e-04, 2.624e-04	0.47
SexMale	3.626e-02	3.897e-02	-3.685e-02, 1.201e-01	0.87
Bleed time	-6.712e-05	3.205e-04	-1.278e-03, 8.659e-04	0.33
Bleed time x SMI	7.968e-08	9.478e-07	-7.024e-06, 1.239e-05	0.03
Bleed time x sex	-2.854e-06	8.008e-05	-7.773e-04, 6.610e-04	0.05

(K) β-OH-butyrate

	Estimate	Unconditional SE	CI (95%)	RI
(intercept)	9.019e-01	0.968	-1.022, 2.826	
StageFuelling	-4.725e-02	0.155	-0.359, 0.264	1
**StagePre-departure**	**0.479**	**0.196**	**8.493e-02, 0.872**	**1**
SMI	-1.452e-03	3.264e-03	-8.010e-03, 5.081e-03	0.96
SexMale	4.947e-02	0.274	-0.802, 1.143	0.29
Bleed time	7.086e-03	7.928e-03	-7.150e-03, 2.443e-02	0.82
SMI x sex	-2.174e-04	9.704e-04	-7.066e-03 2.075e-03	0.09
Bleed time x sex	-2.174e-04	9.704e-04	-5.625e-03 4.540e-03	0.02

Appendix S2

Fig. S1 Relationships between oxidative status and cytochrome c oxidase (COX) at wintering (A), fuelling (B), and pre-departure (C) stages. Note that relationships for the wintering stage are also shown in Fig. 4 of the main text and are the only significant ones (see *Results* for further details).

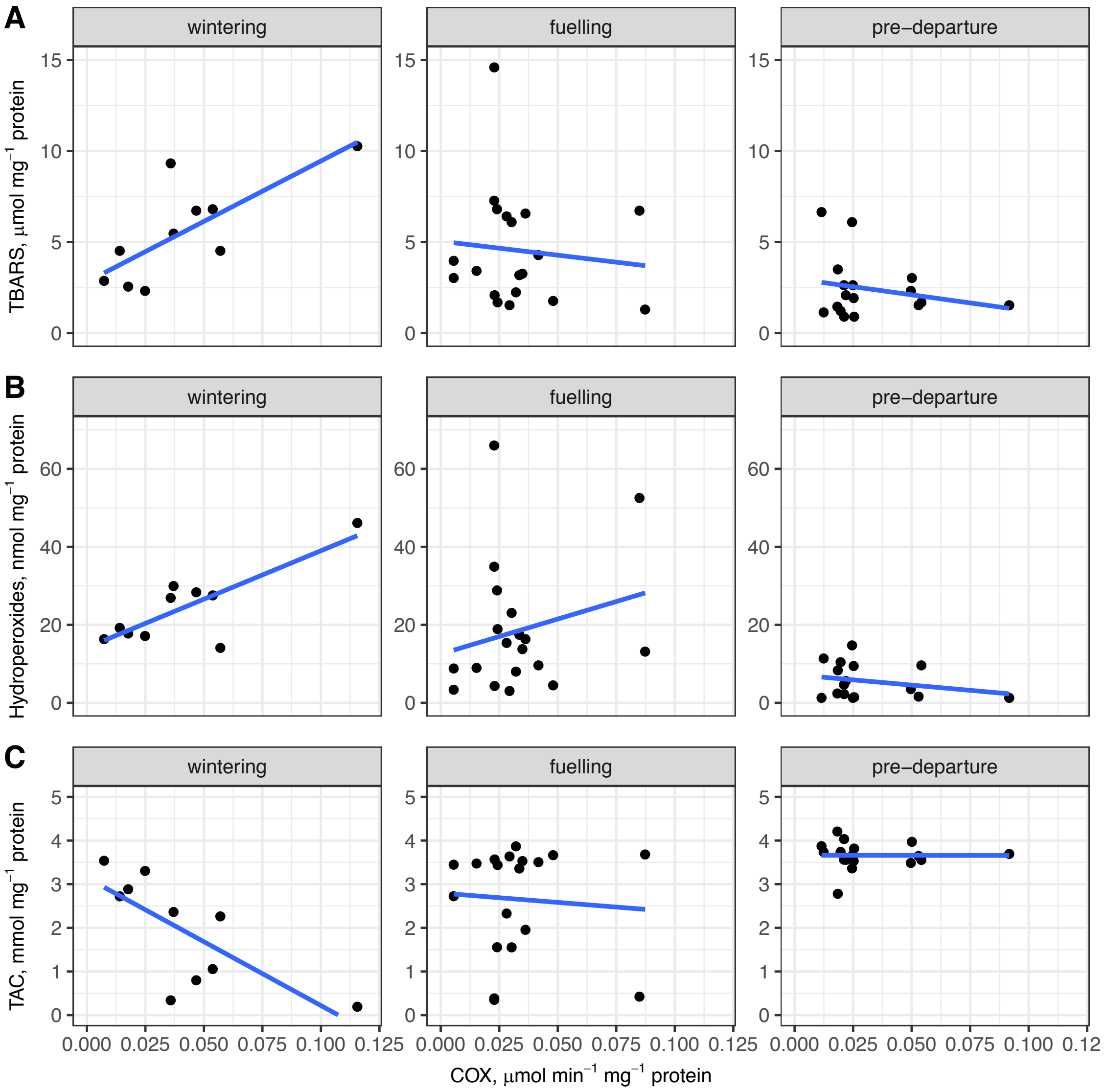

Fig. S2 Relationships between oxidative status and citrate synthase (CS) at wintering (A), fuelling (B), and pre-departure (C) stages. Note that relationships for the wintering stage are also shown in Fig. 4 of the main text and are the only significant ones (see *Results* for further details).

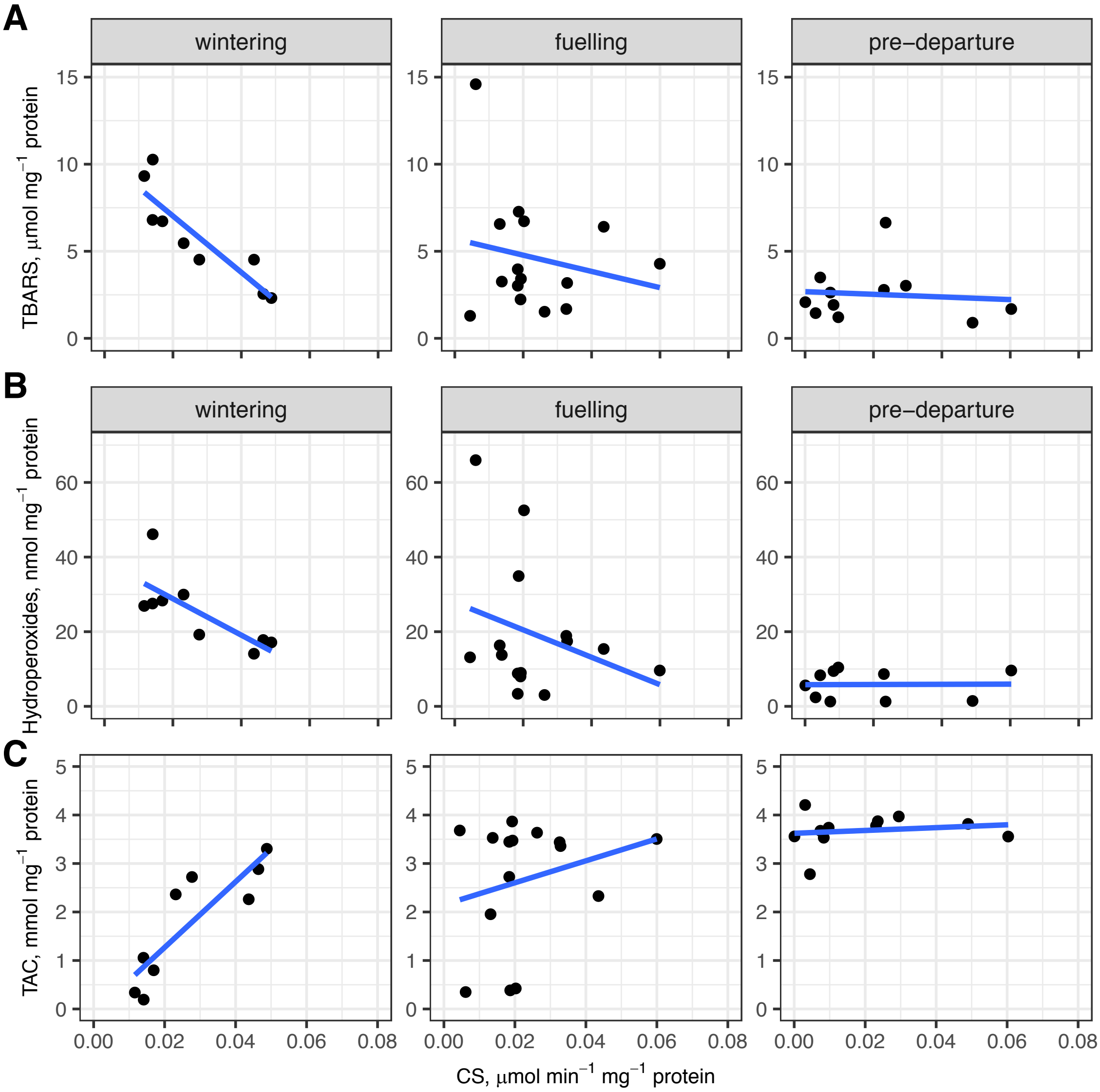

Supplement: Supplementary file 1 — Supplementary information [file 41598_2019_54057_MOESM1_ESM.pdf]
